# Lentivirus vector-mediated mitofusin-2 overexpression in rat ovary changes endocrine function and promotes follicular development *in vivo*

**DOI:** 10.3892/etm.2014.1835

**Published:** 2014-07-09

**Authors:** XIAOJING HU, XIUBING LEI, JIDONG WANG, HONGJUAN PAN, CONG LI, ZHENWEI YAO

**Affiliations:** 1 Department of Obstetrics and Gynecology, The First Affiliated Hospital of Chongqing Medical University, Chongqing 400016, P.R. China; 2 Department of Infectious Disease, The People’s Hospital of Chongqing Banan, Chongqing 401320, P.R. China; 3 Department of Obstetrics and Gynecology, Jinan Central Hospital, Jinan, Shandong 250013, P.R. China; 4 Department of Obstetrics and Gynecology, The Third People’s Hospital of Wuhan, Wuhan, Hubei 430060, P.R. China

**Keywords:** fluorescence microscopy, lentivirus vectors, mitofusin-2, overexpression, radioimmunoassay, western blotting

## Abstract

The aim of the present study was to evaluate the expression and effect of rat mitofusin-2 (rMfn2) in the ovaries and other organs in rats. Rat models were developed by the intraovarian microinjection of an rMfn2-overexpressing lentiviral vector. Lenti-green fluorescent protein (GFP)-rMfn2 was microinjected into rat ovaries at a dosage of 2×10^6^ tuberculin units virosome (n=25) and lenti-GFP was microinjected as a control (n=25). The expression of rMfn2 in the ovaries and other tissues was observed by fluorescence microscopy on days 7, 15, 30, 45 and 60 after the microinjection (n=5/day from each group). The serum levels of estradiol (E_2_), progesterone (P), follicle-stimulating hormone (FSH) and luteinizing hormone (LH) were determined by radioimmunoassay. Western blotting was used for the quantitative analysis of the expression of rMfn2 and the progesterone receptor (PR), estradiol receptor (ER), luteinizing hormone receptor (LHR) and follicle-stimulating hormone receptor (FSHR). The expression of rMfn2 was detected on day 7 after infection, increased with time and was maintained efficiently until day 60. In addition, rMfn2 was highly expressed in the fallopian tubes, uterus, cardiac muscle, liver and kidney, but expressed at a low level in adipose tissue. The serum levels of E_2_ and P in the model group were significantly increased compared with those in the control group, whereas the FSH and LH levels showed no significant difference between groups. The expression levels of the ER and PR in the model group were higher than those in the control group; however, no significant difference was observed between groups for the expression levels of LHR and FSHR. These findings suggest that the intraovarian microinjection of lenti-GFP-rMfn2 resulted in a significant time-dependent overexpression of rMfn2 in various organs, and that rMfn2 overexpression in rat ovaries changed the endocrine function and promoted follicular development.

## Introduction

Mitofusin-2 (Mfn2), also named hyperplasia suppressor gene (HSG), is a transmembrane GTPase embedded in the mitochondrial outer membrane that mediates mitochondrial fusion (1). Mfn2 deficiency and mutations have been linked to human neurodegenerative diseases, including Charcot-Marie-Tooth type 2A and other hereditary motor and sensory neuropathies (2–4). A number of studies have shown that Mfn2 regulates both mitochondrial fusion and mitochondrial apoptotic signaling and is involved in the pathogenesis of disease conditions such as obesity, type 2 diabetes, insulin resistance and the survival of different epithelial cancer cell lines (5–11). However, to the best of our knowledge, there are no published studies concerning the involvement of Mfn2 in the reproductive system. A study demonstrated that mice that are deficient in either Mfn1 or Mfn2 die early in the embryonic stages of development, probably due to underlying mitochondrial defects (5). Emerging evidence indicates that Mfn2 is characterized as a cell proliferation inhibitor, remarkably suppressing the injury-mediated proliferation of vascular smooth muscle cells with a potential apoptotic effect via the mitochondrial apoptotic pathway (12,13). A further study has shown that the overexpression of Mfn2 inhibits hepatocellular carcinoma cell proliferation and induces apoptosis via Bax, and adenovirus-mediated Mfn2 upregulation significantly suppresses the growth of subcutaneous tumors in nude mice both *in vivo* and *in vitro* (14). In addition, Mfn2 has the comparable antiproliferative effects in different types of malignancies that occur in the lungs, liver, breast, colorectal system and urinary bladder (6,15–17). At present, there are a large number of studies about Mfn2, but few of these are morphological studies.

Currently, the involvement of Mfn2 in the reproductive system has not been well studied, and most of the studies concerning the Mfn2 gene have been conducted using adenoviral vectors or lentiviral vectors, as well as cells infected with virus and implanted into nude mice. There have been few studies concerning gene transfection into normal organs *in vivo*. In the present study, lentiviral vector-mediated rat Mfn2 (rMfn2) gene was used to transfect rat ovaries. The effectiveness of the transfection was verified, and the expression and effect of rMfn2 was observed in the rat ovaries and other organs.

## Materials and methods

### Animals

Fifty female Sprague-Dawley rats (2 months old, weighing 180–200 g) were purchased from the Experimental Animal Center of Chongqing Medical University (Chongqing, China). The rats were divided randomly into two groups: lenti-GFP (green fluorescent protein)-rMfn2 and lenti-GFP. Both groups received lentivirus-mediated gene transfer *in vivo*. Rats in the two groups were further divided into the following five groups: 7, 15, 30, 45 and 60 days (n=5). In addition, five female Sprague-Dawley rats (2 months old, weighing 180–200 g) were purchased to be used as a blank control group (uninfected group). All animal experiments were conducted in accordance with the guidelines of the Animal Care and Use Committee of Chongqing Medical University (Chongqing, China). All surgical interventions and postoperative animal care were carried out in accordance with the Guide for the Care and Use of Laboratory Animals (National Research Council, Washington, DC, USA, 1996).

### Microinjection of lentiviral vectors into rat ovaries

A lentivirus encoding the complete rMfn2 open reading frame (lenti-GFP-rMfn2) and a control lentivirus encoding GFP open reading frame (lenti-GFP) were constructed by Western Biotechnology Inc. (Chongqing, China). Anesthesia was induced with 10% chloral hydrate at a dose of 100 mg/kg by intraperitoneal injection. Following anesthesia, all rats were weighed prior to surgery. Then, the rats were placed in a prone position and a microtubule incision of ~1.0–2.0 cm was made on the surface of the back to the right of the position of the ovary. The hypoderma, muscular layer and peritoneum were cut and the ovary was drawn out from the abdominal cavity. Approximately 10 μl of the lentiviral vector solution (either lenti-GFP or lenti-GFP-Mfn2, titer at 2×10^8^ tuberculin units/ml) was slowly microinjected into the sub-envelope of the ovary.

### Sample collection

All rats in the two groups were sacrificed to collect organs on day 7, 15, 30, 45 and 60 following the microinjection. The bilateral ovaries, uterus, hearts, livers and kidneys of the rats were excised, blood specimens were extracted from the heart, and the serum was cryopreserved at −80°C. Certain specimens from the two groups were fixed with 4% paraformaldehyde, whereas the remaining specimens were placed in liquid nitrogen for 2 h and then stored at −80°C until further use.

### Radioimmunoassay

The concentrations of the serum gonadal hormones luteinizing hormone (LH), follicle-stimulating hormone (FSH), estradiol (E_2_) and progesterone (P) were determined using commercial double-antibody radioimmunoassay kits (Pu’er Biotechnology, Beijing, China) in order to assess the endocrine changes in the rats.

### Fluorescence microscopy

The presence and location of rMfn2 protein was determined by an inverted fluorescence microscope (IX-71, Olympus, Tokyo, Japan). Frozen and paraffin-imbedded sections were prepared for fluorescence microscopy for every group. The green fluorescence densities for organ sections from each group were calculated and analyzed for statistical differences.

### Western blotting

At 45 days after transfection, the expression of rMfn2 in various organs was determined by western blotting. Antibodies of the luteinizing hormone receptor (LHR), follicle-stimulating hormone receptor (FSHR), estradiol receptor (ER) and progesterone receptor (PR) were purchased from Beijing Biosynthesis Biotechnology Co., Ltd. (Beijing, China). Proteins were extracted with ice-cold radioimmunoprecipitation assay buffer containing 1 mM phenylmethyl sulfonylfluoride according to the manufacturer’s instructions (Beyotime Institute of Biotechnology, Haimen, China). Total proteins (50 μg) were separated on 10% sodium dodecyl sulfate polyacrylamide gel electrophoresis gel and transferred to polyvinylidene fluoride membranes, which were blocked by Tris-buffered saline Tween-20 containing 5% skimmed milk at room temperature for 2 h prior to being incubated with primary antibodies, specifically, rabbit polyclonal antibody against rMfn2 (1:500) (Abcam, Cambridge, UK) and rabbit anti-β-actin (1:1,000) (Sigma-Aldrich, St. Louis, MO, USA) at 4°C overnight. After washing three times with Tris-buffered saline Tween-20, the membranes were incubated with secondary antibody (horseradish peroxide-labeled anti-rabbit antibody, 1:5,000) for 1 h at room temperature. The color reaction was detected using chemiluminescence (BeyoECL Plus, Beyotime Institute of Biotechnology, Haimen, Jiangsu, China), and the results were analyzed using a professional GelDoc2000 gel imaging system (Bio-Rad, Hercules, CA, USA).

### Statistical analysis

All statistical analyses were performed with SPSS version 17.0 for Windows (SPSS Inc., Chicago, IL, USA). Data are presented as means ± standard error of the mean (SEM). Statistical differences were assessed by one-way ANOVA. P<0.05 was considered to indicate a statistically significant difference.

## Results

### Rat models are successfully developed by the intraovarian microinjection of rMfn2-overexpressing lentiviral vector

**Figure 1:**
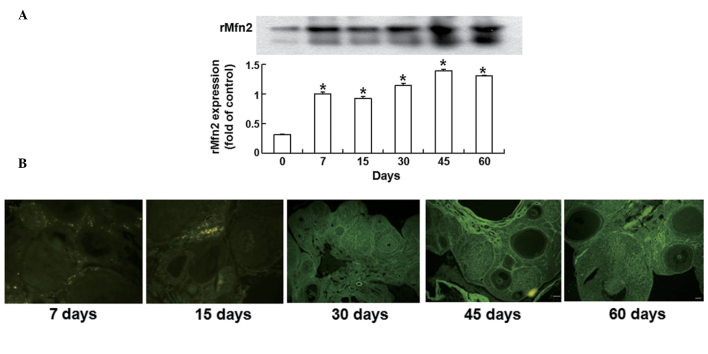
Expression of rMfn2 protein in rat ovary after infection. (A) Time-dependent overexpression of rat mitofusin-2 (rMfn2) in ovaries determined by western blotting. Ovaries were collected from rats on days 0, 7, 15, 30, 45, and 60 after infection with an rMfn2-overexpressing lentiviral vector. Quantitative data are means ± SEM (n=5 independent experiments).^*^P<0.01 vs control group. Rats on day 0 were used as control. (B) Fluorescence images showing the expression of rMfn2 in rat ovaries on days 0, 7, 15, 30, 45 and 60 after transfection. Magnification, ×100. Ovarian morphology was shown using fluorescence microscopy. rMfn2 expressed in follicles of various development stages and in fresh corpora lutea. Scale bars, 100 μm.

To examine the infection effectiveness of rMfn2 in the ovaries, Western blotting analysis was conducted. Ovaries were collected from the rats on days 0, 7, 15, 30, 45 and 60 after infection. rMfn2 protein expression was significantly increased on day 7 following the intraovarian microinjection of lenti-GFP-rMfn2 into the rat ovaries compared with those in the uninfected group (P<0.01). As the time was prolonged, rMfn2 expression increased gradually, reached a maximum on day 45 and remained stable until day 60 following infection (Fig. 1A). These results demonstrate that the rat model was successfully developed by the intraovarian microinjection of an rMfn2-overexpressing lentiviral vector.

### Overexpression of rMfn2 in rat ovary changes endocrine function and promotes follicular development

To detect changes in ovarian morphology and hormonal changes following lentiviral microinjection, fluorescence microscopy and radioimmunoassays were used, respectively. Fluorescence images on day 30 after infection showed that the ovaries from the lenti-GFP-rMfn2 group had few primary follicles and many fresh corpus lutea (Fig. 1B). As the infection time was prolonged, the ovaries in the lenti-GFP-rMfn2 group exhibited follicles in various stages of development, including secondary follicles, Graafian follicles and fresh corpora lutea. On day 60 after infection, developed secondary follicles and fresh corpora lutea remained visible in the ovaries (Fig. 1B). Radioimmunoassay showed that the serum hormonal concentrations in the rats on days 30, 45 and 60 after infection differed between the lenti-GFP-rMfn2 group and the lenti-GFP group. The levels of P and E_2_ in the model group were significantly higher than those in the control group (P<0.01), whereas no significant differences in FSH and LH levels were observed between the two groups (P>0.05; Table I). These data indicate that rMfn2 overexpression in rat ovaries altered the endocrine function and promoted follicular development.

### Expression of LHR, FSHR, ER and PR is consistent with the hormonal changes

**Figure 2:**
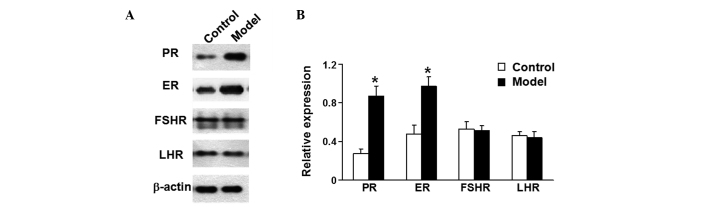
Relative expression of ER and PR in the uterus and FSHR and LHR in ovary tissue. (A) Western blot analysis for ER, PR, FSHR and LHR. β-actin was used as reference. Control, lenti-GFP group; Model, lenti-GFP-rMfn2 group. (B) Quantification of the relative expression of ER, PR, FSHR and LHR. Data are means ± SEM (n=5 independent experiments). ^*^P<0.01 vs. the control group. ER, estrogen receptor; PR, progesterone receptor; FSHR, follicle-stimulating hormone receptor; LHR, luteinizing hormone receptor; GFP, green fluorescent protein; rMfn2, rat mitofusin-2.

To examine the expression of LHR, FSHR, ER and PR, western blotting was performed. PR and ER protein expression levels in the uterus were higher in the model group rats than in the control group (P<0.05; Fig. 2). However, the FSHR and LHR expression levels in the ovaries of both groups of rats showed no significant difference between the groups (P>0.05; Fig. 2). These data demonstrate that the expression levels of LHR, FSHR, ER and PR were consistent with the observed hormonal changes.

### Expression of rMfn2 in other organs differs between groups on day 45 after infection

**Figure 3:**
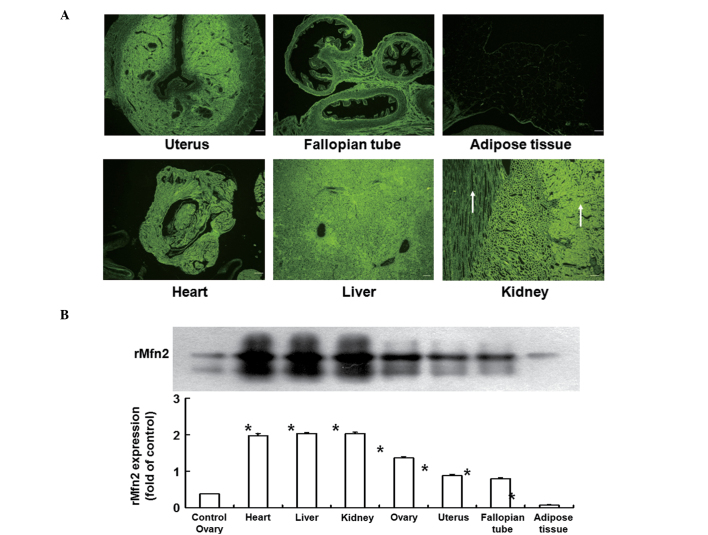
Expression of rat mitofusin-2 (rMfn2) in various organs and tissues of rats on day 45 after transfection. (A) Fluorescence images of various organs and tissues of rats. White arrow on the left, renal cortex; white arrow on the right, renal pyramids. Scale bars, 100 μm. Magnification, ×100. (B) Western blotting for the detection of rMfn2 protein levels in various organs after infection. Quantitative data are means ± SEM (n=5 independent experiments). ^*^P<0.01 vs. control group.

To determine the expression of rMfn2 in other organs of the rats, fluorescence microscopy and western blotting were used. After the intraovarian microinjection of the rMfn2-overexpressing lentiviral vector, green fluorescence was detected not only in ovarian tissues but also in other tissues and organs. On day 45 after infection, paraffin sections of the organs were observed under a fluorescence microscope. In the uterus, it was observed that the fluorescence density in the endometrial layer was stronger than that in myometrium and serosa, but weaker than that in endometrial epithelial cells and endocrine glands (Fig. 3A). By contrast, green fluorescence was evenly expressed in the fallopian tubes, adipose tissue, cardiac muscle, liver and kidney, but the fluorescence density in adipose tissue was weaker than that in other organs (Fig. 3A). In addition, the fluorescence intensity gradually decreased from the renal cortex to the renal pyramids in the kidney (Fig. 3A). Consistent with the results of fluorescence images, western blotting analysis showed that rMfn2 expression in the fallopian tubes, uterus, cardiac muscle, liver and kidney was significantly increased compared with that in control ovary (P<0.01), whereas rMfn2 expression in adipose tissue was significantly lower than that in control ovary (P<0.01; Fig. 3B). These data indicate that the expression of rMfn2 in other organs of rats was varied on day 45 after infection.

## Discussion

In this study, rat models were developed through the intraovarian microinjection of an rMfn2-overexpressing lentiviral vector in order to detect the effect and expression profile of rMfn2 in rats. It was observed that even though the lentiviral vector was microinjected into the sub-envelope of the ovary, the fluorescence density in the ovary was enhanced with the prolongation of time and an efficient and strong expression was maintained until day 60 after infection. Western blotting analysis was consistent with this, demonstrating that rMfn2 expression in the ovary increased gradually, reached a maximum on day 45 and was maintained stably until day 60 after infection. This suggests that the overexpression of rMfn2 in the rat ovary was successfully induced by the intraovarian microinjection of the lentiviral vector, and that lentiviral vector-mediated exogenous genes can be expressed stably in the ovary *in vivo*.

In addition, after transfection of the rMfn2-overexpressing lentiviral vector into the rat ovary, the ovarian morphology was observed under a fluorescence microscope. Fluorescence images showed that rMfn2 was expressed in ovarian follicles, cortex, stroma and corpus luteum. In the lenti-GFP-rMfn2 group, the ovary exhibited follicles in various stages of development, including secondary follicles, Graafian follicles and fresh corpora lutea. On day 60 after infection, developed secondary follicles and fresh corpora lutea remained visible in the ovaries, suggesting that Mfn2 may be involved in the growth and development of follicles. Furthermore, serum hormonal changes in the rats of the two groups were studied 30, 45 and 60 days after infection. P and E_2_ levels in the model group were markedly higher than those in the control group, whereas no significant difference was observed in FSH and LH levels between the two groups. In addition, western blot analysis showed that PR and ER protein expression levels in the rat uterus in the model group were higher than those in the control group, but no significant difference existed in the FSHR and LHR expression levels in rat ovaries between the two groups. By consideration of published data and the results of the present study, it is hypothesized that Mfn2 plays an important role in the development of follicles and improves ovarian endocrine function. However, the mechanism remains to be identified.

The initial aim of the present study was to test the effect of rMfn2 on the functioning of the ovary, so intraovarian injection was selected. Notably, it was observed that rMfn2 was also expressed in the uterus, fallopian tubes, adipose tissue, cardiac muscle, liver and kidney. Consistent with a previous study, rMfn2 was observed to be highly expressed in the ovary, uterus, fallopian tubes, cardiac muscle, liver and kidney, but its expression level in adipose tissue was low (13). Therefore, the data of the present study may be interpreted to suggest that the lentivirus spread through the blood to induce the overexpression of rMfn2 in multiple organs. These data present a new method for the injection of viruses in animal research. In addition, the present study found that rMfn2 expression in different parts of certain organs was varied. In the kidney, the fluorescence intensity was gradually weakened from the renal cortex to the renal pyramids. In the uterus, the fluorescence density in the endometrial layer was stronger than that in the myometrium and serosa, but weaker than that in endometrial epithelial cells and endocrine glands. These results provide a basis for future studies on animal diseases.

Moreover, a previous study indicated that adenoviral gene transfer of rMfn2 inhibited the mitogenic stimuli-mediated proliferation of cultured Wistar-Kyoto vascular smooth muscle cells, blocked balloon injury-induced neointimal vascular smooth muscle cell proliferation and restenosis *in vivo*, and had a potent antiproliferative effect in a variety of cancer cell lines, particularly the breast cancer cell line BM-1 (13). Another study confirmed the antitumor activity of an adenoviral vector encoding human Mfn2 (designated Ad5-hHSG) in a variety of cancer cell lines (6). These data indicate that Ad5-hHSG has the potential to be a gene therapeutic drug. In another study, the results indicated that hMfn2 inhibited tumor cell proliferation and increased the sensitivity of tumor cells to chemotherapy (18). It has also been suggested that Ad5-hHSG can increase the radiosensitization and chemosensitization of A549 and HT-29 cells to a level even higher than that by Ad5-P53 (6). At present, multiple therapies are used to treat cancers. The intratumoral injection of Ad-P53 (INGN201) in combination with radiation therapy is well tolerated and shows evidence of causing regression of the primary injected tumor (19). By combining these reports with the results of the present study, it is suggested that a new therapeutic approach may be developed based on *in vivo* Mfn2 overexpression via gene transfer. A special vehicle carrying the human Mfn2 gene may be injected into specific organs, and stably inhibit tumor growth and metastasis in the long term. This approach only requires a single injection, which is minimally invasive. Although certain studies have indicated that recombinant vectors have no cytotoxicity to animals *in vivo* (20,21), to the best of our knowledge there have been no related studies on the human body.

In summary, rat models were developed through the intraovarian microinjection of an rMfn2-overexpressing lentiviral vector. The intraovarian microinjection of lenti-GFP-rMfn2 resulted in a significant time-dependent overexpression of rMfn2 in various organs of rats. Overexpression of rMfn2 in the rat ovary promoted the development of follicles and improved ovarian endocrine function.

## Figures and Tables

**Table I:** Comparison of the serum levels of hormones between the model and control groups.

Groups	N	FSH (MIU/m)	LH (MIU/ml)	P (ng/ml)	E_2_ (pg/ml)
Control	15	1.18±0.51	4.79±0.35	4.54±1.63	3.76±1.53
Model	15	1.42±0.34	4.91±0.38	18.51±3.43	22.94±2.44
P-value	-	0.137	0.380	<0.001	<0.001

Data are presented as mean ± standard error of the mean of the values on days 30, 45 and 60 post-lentiviral microinjection. P<0.05, significant difference between the two groups. FSH, follicle-stimulating hormone; LH, luteinizing hormone; P, progesterone; E_2_, estradiol; control, lenti-GFP group; model, lenti-GFP-rMfn2 group; GFP, green fluorescent protein; rMfn2, rat mitofusin-2.
